# Cadaveric Stem Cells: Their Research Potential and Limitations

**DOI:** 10.3389/fgene.2021.798161

**Published:** 2021-12-22

**Authors:** Julia Cieśla, Marcin Tomsia

**Affiliations:** ^1^ School of Medicine in Katowice, Medical University of Silesia, Katowice, Poland; ^2^ Department of Forensic Medicine and Forensic Toxicology, Medical University of Silesia, Katowice, Poland

**Keywords:** cadaveric scaffolds, cadaveric stem cells, regenerative medicine, transplantology, thanatotranscriptome

## Abstract

In the era of growing interest in stem cells, the availability of donors for transplantation has become a problem. The isolation of embryonic and fetal cells raises ethical controversies, and the number of adult donors is deficient. Stem cells isolated from deceased donors, known as cadaveric stem cells (CaSCs), may alleviate this problem. So far, it was possible to isolate from deceased donors mesenchymal stem cells (MSCs), adipose delivered stem cells (ADSCs), neural stem cells (NSCs), retinal progenitor cells (RPCs), induced pluripotent stem cells (iPSCs), and hematopoietic stem cells (HSCs). Recent studies have shown that it is possible to collect and use CaSCs from cadavers, even these with an extended postmortem interval (PMI) provided proper storage conditions (like cadaver heparinization or liquid nitrogen storage) are maintained. The presented review summarizes the latest research on CaSCs and their current therapeutic applications. It describes the developments in thanatotranscriptome and scaffolding for cadaver cells, summarizes their potential applications in regenerative medicine, and lists their limitations, such as donor’s unknown medical condition in criminal cases, limited differentiation potential, higher risk of carcinogenesis, or changing DNA quality. Finally, the review underlines the need to develop procedures determining the safe CaSCs harvesting and use.

## Introduction

Stem cells (SCs) play a significant role in modern regenerative medicine. These undifferentiated, self-renewing cells forming populations show multi-line differentiation potential (Sharma, 2018). Embryos are a rich source of pluripotent stem cells (PSCs) that differentiate into all three germ layers: endoderm, mesoderm, and ectoderm (Bar and Benvenisty, 2020). Additionally, PSCs can incorporate into blastocysts, indicating the possibility of creating chimeric embryos and xenogenic organogenesis development (Fu et al., 2020). However, using human embryonic stem cells (ESCs) is ethically controversial, and the moral status of such procedures is undefined yet (Sawai et al., 2020). Similar controversies raise fetal stem cells (FCSs), considered the secondary source of stem cells (Liska et al., 2017). Stem cells can be sourced from somatic cells of adult organisms as it is possible to reprogram them to the pluripotent state. The cells sourced this way are called induced pluripotent stem cells (iPSCs). Yu et al. proved that reprogramming is possible using only four transcription factors: OCT4, SOX2, NANOG, and LIN28 (Yu et al., 2007). The important feature of somatic (tissue) stem cells, and the progenitor cells formed from them, is the possibility of neoplastic transformation. They may become cancer stem cells able to further proliferation and *in vivo* tumor formation (Bjerkvig et al., 2005; Soltysova et al., 2005).

Despite being very promising, the iPSCs/ESCs therapy is characterized by high culture costs and restrictive legislative protocols (Perán et al., 2013). The problem with using somatic stem cells is, on the other hand, the low number of donors (Strässler et al., 2018). Since recent studies report on the newest therapeutical stem cells applications, growing numbers of SCs transplantations, and expanding possibilities of regenerative medicine, it became necessary to search for alternative sources of stem and progenitor cells (Condic and Rao, 2010). Human cadaver stem cells (CaSCs) may serve as such source (De Pauw et al., 1998). Recent years were abundant in studies on sourcing, harvesting, isolating, and using cadaver stem and progenitor cells. So far, mesenchymal stem cells, neural stem cells, retinal stem cells, adipose stem cells, and induced pluripotent stem cells were successfully sourced from human and animal cadavers (Bliss et al., 2012; Mansilla et al., 2015; Saito et al., 2020; Liu et al., 2021).

Human body has been used for medicine development after death for ages. Teaching anatomy using human cadavers has been a standard for centuries (Bahşi et al., 2021). Organ transplantology uses human organs harvested postmortem. The first kidney transplant was done by Joseph Murray, Hartwell Harrison, and John Merril in the late 1960s. The 1970s start era of the heart and lungs (Hardy, 1999; Tan and Linskey, 2019) and liver transplants (Krawczyk, 2018). Nowadays, these techniques are carried out globally with increasing success. The idea of transplanting hematopoietic cells emerged in the 20th century. However, the first bone marrow transplant took place in 1998 and was done by Polish scientists Jan Stefan Raszek and Franciszek Groer from the University of John II Casimir in Lviv (Kobus and Małyszko, 2015).

Nowadays, regenerative medicine and transplantology expand their possibilities to using materials collected postmortem. The simple protocol for collecting and storing the skin from dead donors makes possible the successive skin transplants in patients with extensive burns, decreasing their mortality or in the therapy of hard-to-heal wounds (Sachkov et al., 2018), including lower limbs ulcers not susceptible to other treatment (Mosti et al., 2020). Additionally, postmortem collected bones, due to keeping their mechanical properties after the death, served as research material in studies evaluating the effect of local bone reinforcement by injecting a three-phase calcium-based implant, the novel osteoporosis treatment (Stroncek et al., 2019). Postmortem collected bone fragments can be frozen up to eight times without losing their morphological and biomechanical properties. Defrosted and unused for allotransplants, bone fragments can be frozen again, saving the materials collected from donors (Shaw et al., 2012).

Taking into account the vast possibilities of using stem cells in novel therapeutic methods and growing numbers of reports on using cadavers bodies in regenerative medicine, makes it essential to investigate more thoroughly the role of stem cells isolated from materials collected postmortem, their properties, and their potential applications in various branches of medicine. The presented review focuses on recent, both *in vitro* and *in vivo*, studies on human and animal cadaveric stem cells (CaSCs). It also discusses the available solutions for constructing the scaffolding for stem cells and other cells transplanted as prosthetics during damaged organs repair. Finally, it summarizes the research on genes expression after death and its influence on cells viability in postmortem environment.

### Mesenchymal Stem Cells (MSCs)

Mesenchymal stem cells (MSCs) are a multipotential group of cells that are a source of various types of connective tissues (Caplan, 1991). International Society for Cell Therapy (ISCT) suggests using the following strict criteria to distinguish the mesenchymal stem cells: the ability to use them in the plastic adherence method (Hurmuz et al., 2016), expression of markers: CD73, CD90, CD105, and lack of expression of markers: CD45, CD34, CD11b/14, CD79α/19, and HLA-DR and the ability to differentiate into osteogenic, chondrogenic and adipogenic lines (Dominici et al., 2006). Additionally, MSCs do not present markers characteristic of the hematopoietic line (Garfias et al., 2012; Valente et al., 2014).

Most frequently, the cadaveric MSCs are isolated from the bone marrow (Mansilla et al., 2015; Korchunjit et al., 2019). They can also be successfully isolated from ligaments (Shikh Alsook et al., 2015), arteries fragments (Valente et al., 2014), the marginal zone of the cornea, and sclera (Rama et al., 2010), and other tissues. Several studies tested the abilities of postmortem collected tissues to produce cell lines characteristic for the MSCs. Studies showed that the fragments of horse ligaments could differentiate into osteocytes, chondrocytes, or adipocytes up to 72 h after collecting them postmortem. The cells showed the presence of CD90, CD105, and CD73 markers. However, if the culture environment did not contain appropriate stimulants, neuronal cells were observed (Shikh Alsook et al., 2015). Korchunjit et al. proved that MSCs isolated from horse bone marrow differentiate into tenocytes (tendon cells) (Korchunjit et al., 2019). Collecting MSCs is possible even 5 years postmortem if the tissues (more specifically, the vessels) are preserved in liquid nitrogen. Apart from showing the presence of mesenchymal markers, the cells showed the presence of CD146, PDGFR-beta, NG2 (pericyte), or nestin (neuronal) markers. Moreover, they showed high proliferation ability, up to 12 passages (Valente et al., 2014). MSCs were also successfully sourced postmortem from the cornea and sclera marginal zone, which contains limbus stem cells, regenerating the cornea, i.e., after the burns (Rama et al., 2010). The cells collected postmortem showed MSCs traits and secreted the immunosuppressants like TGFbeta1 affecting lymphocytes B receptors (TCR). This trait increases the value of MSCs for regenerative medicine applications (Garfias et al., 2012). So far, the most attention was given to immunosuppressive features of MSCs isolated from the bone marrow, including their ability to hinder the contact between the naïve lymphocytes T and memory cells and their related antigens (Krampera et al., 2003), and changes from lymphocyte activity to allogeneic cells and target tissues (in the case of studies on baboons) (Bartholomew et al., 2002). Since the cells secrete bioactive factors, e.g., acting as immunomodulators, it is suggested to change the commonly used name of MSCs (Caplan, 2017).

Mesenchymal stem cells were also isolated postmortem from the synovium (De Bari et al., 2001a; De Bari et al., 2008) and the periosteum (De Bari et al., 2001b; De Bari et al., 2008). Cells from both sources differentiated into the MSCs lines. Moreover, the cells originating from the synovium differentiated sporadically into the myogenic line (De Bari et al., 2001a). In the case of the cells originating from the periosteum, no differences were found between cells from living donors (after knee surgery) and deceased donors (up to 12 h postmortem) (De Bari et al., 2001a). It is important to mention here the *in vivo* studies involving the MSCs originating from the periosteum and synovium and their subcutaneous implantation using osteoinductive scaffolds in mice. The study showed that the cells originating from the periosteum have a greater potential for bone formation than those originating from the synovium due to significant phenotypic and differentiation ability differences. Additionally, the researchers developed a mathematical model based on the expression of biomarkers: collagen I and osteoprotegerin (OPG), which aimed to examine the osteogenic potential of the cells regardless of the age of the donor and the origin of the tissue (De Bari et al., 2008). On the other hand, donor age plays a role in the differentiation of cells originating from the periosteum into the chondrogenic line. The spontaneous chondrogenesis in donors over 30 years of age is lost, but it can be induced with TGFb1 and micromass culture (De Bari et al., 2001b). Tissues such as the periosteum and synovium, obtained from deceased donors up to 12 h postmortem, are a promising source of MSCs. Radtke et al. proved that MSCs collected postmortem from the bone marrow of dead horses differentiate much slower than when collected from muscle, periosteum, or adipose tissue. Moreover, they showed that MSCs originating from muscle and periosteum have a higher osteogenic potential than those isolated from adipose tissue and bone marrow (Radtke et al., 2013).

Mesenchymal stem cells from deceased donors could become a valuable tool in modern human and animal regenerative medicine. Mesenchymal stem cells isolated from the horse’s bone marrow could be an excellent source of cells for allogeneic transplants in racehorses suffering from osteoarthritis (Korchunjit et al., 2019). The post-transplant results indicated no side effects, a lower level of osteophyte and mononuclear cell formation in the synovial fluid, and better horse performance in competitions (Korchunjit et al., 2019). Mesenchymal stem cells collected and isolated *in vitro* postmortem from human bone marrow could be used in patients with skin burns or necrotic lesions caused by radiation. The patient with skin burns covering 60% of the body was successfully treated with MSCs transplants that over time began to resemble natural-looking human skin (Mansilla et al., 2015). The triple application of MSCs to a patient with post radiation necrotic leg lesions resulted in a significant improvement of skin condition and the patient’s general condition. Significantly, each MSCs application reduced inflammation. Three months after the first MSCs application, the levels of beta-integrin and CRP (C-reactive protein) decreased to the reference values, which proved the immunomodulatory features of MSCs (Portas et al., 2016). Other studies indicate that MSCs collected postmortem from the human eye and platelet-derived growth factor on (PDGF) secreted by them have a neuroprotective effect on retinal ganglion cells (RGC), which if damaged factor into the optic neuropathy development (Osborne et al., 2018).

### Adipose Delivered Stem Cells (ADSCs)

Stem cells derived from adipose tissue also retain their multipotentiality and can differentiate into many types of cells. The International Fat Applied Technology Society recommends using the term adipose delivered stem cells (ADSCs) for multipotent cells isolated from adipose tissue, showing the ability to adhere to plastic (Gimble et al., 2007), similar to MSCs. Additionally, they should express the following stem cell markers: CD13, CD29, CD44, CD105 and CD166 and transcription factors: Nanog, Oct-4, Sox-2 and Rex-1 (Zhu et al., 2008). Moreover, they should have cytokine secretion properties similar to MSCs from bone marrow, and with the presence of relevant factors, they should promote hematopoiesis and vasculogenesis (Kilroy et al., 2007). Despite the great attention devoted to multipotent bone marrow cells, ADSCs are more frequently used as a source of stem cells for regenerative medicine due to a large number of cells per unit of collected material and low invasiveness of harvesting procedures from living donors (Harasymiak-Krzyżanowska et al., 2013) which are liposuction or needle biopsy (Bunnell et al., 2008). It is worth noting that the number of obtained pre-adipocytes depends on the patient. Also, it depends on the patient’s age, which may limit the following cell differentiation because the differentiation capacity of the ADSCs decreases with the donor’s age (Bunnell et al., 2008).

CD34, also found in vascular endothelial cells, has been suggested as an appropriate marker of ADSCs. The analysis of the CD31 marker, which is specific only to vascular endothelial cells, helps distinguish these two types of cells. The study, examining the most frequent location of ADSCs cells [CD34 (+) CD31 (-)] in cadavers, found that they are abundant in the lower abdomen and thoracic back (Kishi et al., 2010). Since these areas are rich in fibrous connective tissue, the highest concentration of ADSCs is detected in the course of connective tissue and perivascularly (Kishi et al., 2010; Di Taranto et al., 2015). The study on the multipotential abilities of ADSCs collected from cadaver’s superficial adipose tissue (SAT), and deep adipose tissue (DAT) showed that ADSCs isolated and then cultured from SAT presented a higher ability to differentiate than ADSCs isolated from DAT (Di Taranto et al., 2015). ADSCs isolated postmortem from myocardial tissue can also differentiate into myocyte-like cells and express contractile proteins. The study showed that this tissue maintains RNA integrity and secretes substances necessary for differentiation up to 12 h postmortem. This new method extends the use of cadaver ADSCs. It enables easily accessible and cheap *in vitro* testing of the toxicity of the cardioactive drugs (Perán et al., 2013). Interestingly, human ADSCs show the unique ability to survive long after the donor’s death. Human ADSCs taken from subcutaneous axillary adipose tissue survived up to 7 days postmortem, while mouse ADSCs survived only 24 h. The difference has been associated with the small size of mice compared to human cadavers, which accelerates the rotting process (Saito et al., 2020).

### Neural Stem Cells (NSCs)

Cadaveric neural stem cells (NSCs) and neural stem cell transplantation techniques are researched mainly for regenerative medicine purposes to repair damage and neurodegenerative lesions of the central nervous system since brain stem cells cannot regenerate damaged areas (Lovell et al., 2006; Gögel et al., 2011; Mikhailov and Sankai, 2018). Stimulating NSCs development may help form new neurons and replace those lost in aging and neurodegeneration processes (Lovell et al., 2006). On the other hand, *in vitro* studies of NSCs enabled us to discover the pathogenesis of many diseases (Lin and Monje, 2017). Recent findings suggest that a reduced number of NSCs may cause schizophrenia (Reif et al., 2006). So, the cadaveric NSCs could also serve as an important source of information on this subject.

The isolation of multipotent cells from the human brain is very promising. Isolated NSCs react to epidermal growth factor and basic fibroblast growth factor creating spheres that *in vitro* differentiate into neurons, astrocytes, and oligodendrocytes (Arsenijevic et al., 2001). It is believed that fetuses are the best source of such cells, but this source raises serious moral and ethical questions (Xu et al., 2003). Nestin is the intermediate fiber used to identify undifferentiated neuroepithelial cells (Quinn et al., 1999). Also, it was shown that a different ratio of profile markers CD15/CD24/CD29 determines the development of the neuronal lines of neural stem cells towards lines of the nerve spine, neural crest, and neuron population (Pruszak et al., 2009). Neural progenitor cells obtained postmortem from the brain after differentiation show positive immunological staining for beta-tubulin, GFAB (glial fibrillary acidic protein) (Laywell et al., 1999), NauN (neuron postmitotic marker), O4 (immature oligodendrocyte marker), and fibroblasts (for connective tissue) (Palmer et al., 2001).

So far, postmortem studies have been conducted mainly on mice, rats, and human cadavers. They aimed to determine the regenerative and proliferative abilities of cadaver NSCs, how their survival is affected by postmortem interval (PMI), and which parts of the nervous system are the richest source of neural stem or progenitor cells (Laywell et al., 1999; Xu et al., 2003; Liu et al., 2006). Xu et al., using the 4- and 3-week-old rats, showed that NSCs could be isolated from the striatal wall, including the subventricular zone of the lateral ventricle of the brain. In the case of very young rats, 1-day- and 7-day-old, it was possible to isolate NSCs from the lateral forebrain (Xu et al., 2003). Studies on NSCs in mice were carried out on cells collected from the spinal cord and the nodal zone of the forebrain that could differentiate into neurons and glial cells (Laywell et al., 1999). The studies on the stem cells isolated postmortem from the inner ear of mice showed that the spheres from the sensory epithelium frequently differentiate into ear hair cells. In contrast, spheres cultured from the spiral ganglion are a good source of neurons and glial cells even when collected 10 days after the animal death (Senn et al., 2007). The research on NSCs collected from human cadavers showed that the progenitor cells obtained from the brain of an 11-month-old and a 27-year-old cadaver after the temporal cortex removal were the most numerous in the brain sections obtained from the hippocampus and the ventricular zone (Palmer et al., 2001).

As mentioned earlier, human fetuses can be a rich but a controversial source of NSCs/NPCs collected postmortem. Stem/progenitor cells obtained from the bodies of aborted fetuses are referred to as fetal cadaveric stem cells. By isolating NSCs from various sections of the spinal cord of aborted fetuses, Liu et al. showed that the most abundant neurospheres originate from cells collected from the lumbar and sacral spinal cord (Liu et al., 2006). Studies on aborted fetuses also showed that neuroepithelial precursor cells, collected from this source, are mitotically active and form spheres of undifferentiated cells in the presence of epidermal growth factor (EGF) or fibroblast growth factor 2 (FGF 2) (Quinn et al., 1999). Marmosets with spinal cord injury were the first subjects of transplants of NSCs isolated from fetuses. The procedure was successful, and the transplanted fetal NGCs differentiated into neurons, oligodendrocytes, and astrocytes, and the spinal function was recovered (Iwanami et al., 2005). In 2015, the NSCs differentiated from fragments of the midbrain of a naturally aborted fetus at 13 weeks of gestation were successfully transplanted to patients with cervical spine injury (Shin et al., 2015).

Some studies assessed the abilities of the collected cells to create neurospheres, and several relationships were discovered. Xu et al. showed that the number of neurospheres is significantly greater in cultures from the material isolated from rats killed shortly after birth than in cultures from the material collected from rats killed in adulthood (3–4 months) (Xu et al., 2003). Laywell et al., in their study on mice, found that as PMI increased, the ability of cells to form neurospheres decreased (Laywell et al., 1999). Senn et al. successfully isolated stem cells from the mouse inner ear even 10 days after death (Senn et al., 2007). The studies on miscarried fetuses showed a similar relationship. Comparing the storage time of 14-week-old fetuses for 2, 6, and 12 h at 4°C, it was found that the ability to form neurospheres decreased over time, and the most significant regression was observed after 12 h and longer (Liu et al., 2006). Longer *in vitro* storage of NSCs cultures is possible after using trypsin and a growth medium containing 10% dimethylsulfoxide (Palmer et al., 2001). Moreover, animal studies unequivocally indicated that the survival of NSCs depends on the size of the organism from which the cells had been harvested. Maximal survival time for NSCs harvested from goats was established for 56 h postmortem and for NSCs harvested from rats for 18 h (Mikhailov and Sankai, 2018). However, another study showed that cells collected from mice, even 140 h postmortem at 4°C, retain their ability to create mitotically active neurospheres and differentiate into neurons and glial cells. This long-term ability to differentiate is explained by their reduced energy requirements and therefore reduced metabolic activity. The second factor contributing to this phenomenon may be the protective effect of the cerebrospinal fluid (CSF), which creates a suitable environment for the NSCs survival (Laywell et al., 1999). Additionally, the survival of NSCs also depends on the diameter of the spinal cord and the rate of postmortem drying related to animal size and metabolism rate (Mikhailov and Sankai, 2018).

Nerve progenitor cells have also been successfully isolated postmortem from the human retina (Mayer et al., 2005).

### Retinal Progenitor Cells (RPCs)

The human eye can be a valuable source of transplantation material after death, not only because of the corneal transplants that have been performed for decades but also because of the presence of progenitor cells. Isolated postmortem and used for degenerative diseases treatment, retinal progenitor cells (RPCs) are a frequent subject of cadaver research in the context of stem and progenitor cells (Klassen et al., 2004; Mayer et al., 2005). However, neural progenitor cells that can differentiate into neurons due to being isolated from the nervous part of the retina, photoreceptors (Mayer et al., 2005), and glial cells (Carter et al., 2007) are also used in degenerative diseases treatment. Retinal progenitor cells also can differentiate into photoreceptors (Aftab et al., 2009). Differentiation into retinal neurons was possible after collecting cells from the retinas of aborted human fetuses, which showed a similar gene expression profile to cells obtained from mouse embryos (Lamba et al., 2006). Postmortem studies on human fetuses also showed the retinal developmental genes (vimentin, KI67, nestin, PAX6, SOX2, HES5, GNL3, OTX2, DACH1, SIX6, and CHX10) already expressed in the first passage (Schmitt et al., 2009). Also, RPCs collected from the fetal retina between 16 and 18 weeks of gestation are characterized by the longest *in vitro* survival (Iwanami et al., 2005). Cells from the human eye were also collected postmortem to study corneal stem cells regulated by the transcription factors SOX2 and P63 (Bhattacharya et al., 2019).

Retinal progenitor cells express many markers mutual with brain progenitor cells (BPCs). However, it is possible to distinguish them as genes: Dach1, Pax6, Six3, Six6, and recoverins are specifically expressed only in RPCs (Klassen et al., 2004). On the other hand, studies conducted on living donor and cadaveric retinas showed that the main problem with RPCs isolation is maintaining their epithelial differentiation properties and the lack of certain stem cells specific properties, hence the limited use of RPCs depending on the donor origin (Frøen et al., 2011). However, RPCs maintain the ability to form retinal cell neurospheres throughout the donor’s life, so the limitation of their use is not the age of the donor but only PMI in the case of cadaveric donors. The study showed that the longer PMI, the time of neurospheres formation increases (Carter et al., 2007). Other research conducted on the cadavers showed that adding the amniotic fluid preparation to the *in vitro* RPCs medium significantly increases the generation rate of retinal progenitor cells (Sanie-Jahromi et al., 2012).

### Induced Pluripotent Stem Cells (iPSCs)

Induced stem cells are cells derived from the body’s somatic cells and reprogrammed to be pluripotent by specific genes introduction. As a result of this process, iPSCs can intensively multiply and induce the formation of all three layers of germ cells: endoderm, mesoderm, and ectoderm. For this reason, they are considered similar to embryonic stem cells (ESCs) (Omole and Fakoya, 2018). Induced pluripotent stem cells represent a relatively new branch of stem cell research, as the first iPSCs isolation took place in 2006. It was done using mouse fibroblasts and the set of four factors: Oct3/4, Sox2, c-Myc, and Klf4. The induced cells showed actual markers of embryonic stem cells (Takahashi and Yamanaka, 2006). Induced pluripotent stem cells are used in many fields of medicine.

It was quickly discovered that fibroblasts, used to differentiate into iPSCs, can also be collected from the deceased of different ages and at different PMI. Meske et al. showed that it is possible to culture fibroblasts from a section of the skin of the abdomen collected up to 48 h postmortem from a 99-year-old donor (Meske et al., 1999). It was also showed that sheep skin stored in normal refrigeration conditions (4°C) could be a source of fibroblasts that can multiply even at 56 h postmortem (Singh et al., 2011). However, these studies did not test the ability of the harvested cells to differentiate into pluripotent stem cells. Such an experiment was done by Blis et al., who examined fibroblasts taken from the dura mater of the brain and scalp of deceased donors and their ability to differentiate into iPSCs. They proved that cells isolated from the dura mater have 16 times better properties for cultivation than those from the scalp, which, as it turned out, proliferate faster. However, in the case of cells collected from the scalp, longer PMI correlated with the decreased ability to grow fibroblasts successfully. The authors concluded that further research is needed to find out which source is more suitable for generating iPSCs (Bliss et al., 2012). Hjelm et al. presented a postmortem model of iPSCs induction from skin fibroblasts of a 75-year-old male. These cells displayed both nuclear and surface markers of pluripotency and differentiated into neurons and glial cells (Hjelm et al., 2011). The discussed-above studies indicate the enormous potential of somatic fibroblasts collected from the cadavers and the possibility of their induction into pluripotent cells that can be used in regenerative medicine.

### Hematopoietic Stem Cells (HSCs)

Hematopoietic stem cells (HSCs) are at the top of the hierarchy of the cells generated during the hematopoiesis process. They are the source of series of multipotent and then oligopotent progenitor cells specific for a given blood cell type, constituting an essential regulatory factor for the whole process (Bryder et al., 2006). For a long time, the presence of HSCs in the cell culture was difficult to confirm, as no marker was discovered as a positive marker unambiguously indicating their presence. Nowadays, CD34 is a marker routinely determined for HSCs, but it might be absent on some cells. Therefore, the CD133 marker serves as an additional tool in distinguishing HSCs (Handgretinger et al., 2003). Moreover, Ziegler et al. proved that vascular endothelial growth factor receptor 2 (VEGFR2, also known as KDR) is a marker that distinguishes HSCs from hematopoietic progenitor cells (HPCs) (Ziegler et al., 1999). Bone marrow is a rich source of HSCs, especially in sites of increased regional hypoxia, which plays a fundamental role in the differentiation of hematopoietic stem cells (Parmar et al., 2007). In the last 50 years, hematopoietic cells were successfully isolated from the fetal liver (Thomas, 1993), peripheral blood (Anasetti et al., 2012), and umbilical cord blood (Rogers and Casper, 2004).

Bone marrow was analyzed for hematopoietic stem and progenitor cells by many researchers. It was proved that HSCs and HPCs isolated from the femoral bone exposed to heat or ischemia could retain their phenotype and the ability to repopulate even 12 h postmortem. Additionally, they remained viable for up to 4 days when collected and stored at 4°C (Michalova et al., 2011). Söderahl et al. showed that the temperature (4 or 20°C) and the storage time (up to 72 h) did not affect the viability of colony-forming progenitor cells collected postmortem from bone marrow (Söderdahl et al., 1998). The factor that affects their short-term viability is an appropriately selected anticoagulant. Heparin was found to have the best anticoagulant properties. Donors are routinely injected with heparin before organ donation, and therefore the blood in the marrow cavities remains fluid and can be easily aspirated. The study also highlighted the importance of the atmospheric air presence (with 20% oxygen content) in the bags with the collected bone marrow and the beneficial effect of the erythrocytes and leukocytes on the survival of progenitor cells: CFU-GM (Granulocyte-Macrophage Colony-Forming Unit) and BFU-E (Erythroid Burst-Forming Units) (Machaliński et al., 2003). Therefore, it can be concluded that donated organs can also serve as a source of hematopoietic cells. Moreover, storing the bone marrow from heparinized donors at 4°C ensures that HSCs are not degraded by reactive oxygen species (ROS) produced by mitochondria and influencing the process of programmed cell death for up to 7 days (Machaliński et al., 2005). Moreover, the cadavers’ vertebral bodies are the richest source of hematopoietic progenitor cells (Rybka et al., 1995) even though bone marrow is more frequently collected from the iliac plate, the ribs, and sternum (Machaliński et al., 2003).

It is widely postulated to extend the allogeneic bone marrow transplant procedure from deceased donors. In 1986, bone marrow was transplanted from the deceased father to a 12-year-old boy with acute myeloid leukemia. The bone marrow was harvested 40 min postmortem, and the procedure was successful, even though the direct cause of the father’s death was the Graft versus Host (GVH) disease (Blazar et al., 1986). T lymphocytes play an important role in the development of GVH disease. Interestingly, the marrow collected from cadaveric donors has, on average, 10% less of these cells than the marrow from living donors. Removing the T lymphocytes from the transplanted material and leaving only hematopoietic cells, together with adequately selected immunosuppressants, may significantly contribute to therapeutic success (Mugishima et al., 1985). In 1998, a 2.5-year-old girl with neurogenic Gaucher disease received a bone marrow transplant collected 30 min postmortem from a 12-year-old HLA-compliant brother who died after severe neck and head trauma. Since no symptoms of GVH disease were observed, the case confirmed that cadaver bone marrow might be an appropriate source of hematopoietic stem cells used for allogeneic transplantation (Kapelushnik et al., 1998), especially that the number of colony-forming cells [CFU-GM, BFU-E, and CFU-GEMM (Colony-Forming Unit—Granulocyte, Erythroid, Macrophage, Megakaryocyte)] is the same in case of living and cadaveric donors (Machaliński et al., 2003).

## Thanatotranscriptome and DNA/RNA Changes Related to Postmortem Interval (PMI)

Modern science has a tremendous amount of information about the gene expression of living organisms. In contrast, less is known about what happens to the human genome after death. For many years, the researchers tried to explain whether gene activity gradually decreases, or conversely, increases over time from the time of death (Javan et al., 2015). However, now it is known that postmortem changes in tissue gene transcription result not from DNA degeneration but from the active change in expression that lasts many days after the end of vital functions of the system (Ferreira et al., 2018). The intensity of DNA degeneration varies between organs: it is more significant in the spleen than in the brain (Williams et al., 2015). Studies showed that an abundance of active transcript is detectable in the material collected from the prostate gland even 5 days after death (Tolbert et al., 2018). Moreover, RNA isolated from cells of body fluids and human myocardium showed integrity up to 24 h postmortem (González-Herrera et al., 2013), and mRNA isolated from the liver was stable up to 48 h postmortem (Javan et al., 2015).

Increased activity of anti-apoptotic factors plays a significant role in such an unusual phenomenon. In the material collected postmortem from rats, the concentration of BCL2, BFAR, BIRC2 genes increased up to 120 h and positively correlated with PMI. Additionally, the p53 DNA and protein repair mechanisms (TP53BP2, TP73, and BAD genes) were not active until some time has elapsed since death. Moreover, the level of the negative apoptosis regulator XIAP increased overtime (Tolbert et al., 2018), which in postmortem studies on the human liver increased its activity 28 times (Javan et al., 2015). Interestingly, studies in mice showed an opposite trend, of BCL2 gene concentration decreasing with time (Noshy, 2021). Moreover, studies on the brain tissue of rats showed that inflammatory response genes are not activated up to 6 h after death (Halawa et al., 2019). Furthermore, studies on the rat liver subjected to heat stress (41°C) reported a significant reduction in the transcripts of pro-inflammatory genes: TNFα and IL-1 and Bcl-2 genes up to 6 h after death (Halawa et al., 2018). The study also reported an increase in caspase-3 expression 1 h after death and then a decrease at 3 and 6 h postmortem when comparing heat stress conditions to room temperature. Additionally, heat stress reduced the mRNA expression of the c-fos protein (Halawa et al., 2018). The genes responsible for autolysis are also activated after death, as this process is correlated with the production of free radicals. In rats, the production of reactive oxygen species (ROS) and reactive nitrogen species (RNS) increases within 4 h after death, then after 6 h decreases slightly to continue to increase after 8 h. The expression of autophagy-related factor genes is phase-specific. The LC3 gene is rapidly expressed up to 2 h, Beclin-1, ACTG7, and ACTG12 up to 4 h after death (Martínez et al., 2019).

The relationship between gene expression and PMI may play a significant role in forensic medicine. It could help to determine the time of death as accurately as possible, even up to an hour (Halawa et al., 2018), from easily accessible tissues, such as skin and subcutaneous adipose tissue (Ferreira et al., 2018). It is possible to determine PMI based on the anti-apoptotic gene expression panel. Studies on mouse liver showed that Casp3 protein expression increases linearly with PMI, Bax expression increases until 18 h and then decreases, while Trp53 expression increases between 3 and 6 h after death and then decreases between 9 and 24 h (Noshy, 2021). Javan et al. (Javan et al., 2015), taking into account these studies and the studies on gene expression of anti-apoptotic factors in rats (Tolbert et al., 2018) and possible interspecies differences (Noshy, 2021), proved that in human liver cells, the expression of the anti-apoptotic genes BAG3, BAK1, BAX, BIRC5, IL10, NAIP, NFKB1, and RIPK2 decreased almost 6-fold. In contrast, the expression of death-domain proteins TNFRSF10A, TNFRSF11B, YNFRSF25, TNFRSAF9, and TNFRSAF9 decreased 3-fold for PMI from 16 to 48 h (Javan et al., 2015).

Studies on human tissues also showed differences in gene expression after death. It was speculated that gene expression in postmortem body fluid cells correlates with PMI. It was concluded based on the finding that the expression of the MYL3 (myosin three light chain) and VEGFA (vascular endothelial growth factor A) genes in the blood and the MMP9 (matrix metalloprotease 9) gene in the pericardial fluid is higher with a PMI exceeding 12 h (González-Herrera et al., 2013). The fact that some genes increase their expression after death provides valuable information not only for criminologists and forensic doctors but also transplantologists (including those studying stem cells) (Javan et al., 2015). Moreover, it was hypothesized that cells harvested after death and neoplastic cells (in the case of less malignant neoplasms) have a similar gene expression profile and that both types of cells are prone to abnormal gene expression and in the case of cadaveric cells resulting from disturbances in control and homeostatic mechanisms (Bordonaro, 2019). However, postmortem studies on gene expression in stem cells are still lacking. Knowledge of the transcriptional changes in cadaveric stem cells (resulting, among others, from postmortem ischemia) may improve tissue harvesting and organ transplant protocols (Ferreira et al., 2018). Perhaps such information would allow to adjust the time of cell collection to the time of gene expression of specific factors related to the multipotency or pluripotency of stem cells and to manipulate them to improve the therapeutic effects of CaSCs transplantation. It is necessary to check how the expression of the genetic material of stem cells works by postmortem oxidative stress and the rapid activation of autophagy genes, and the rapid activation of various types of apoptotic genes.

## Cadaveric Scaffolds

The cadavers also find use as a scaffold for transplanted stem cells in regenerative medicine. They are used as an environment for stem cells proliferation, growth, and differentiation under *in vivo* culture conditions and as a matrix allowing, among other things, the proper angiogenesis process. To adequately fulfill its role, such scaffold should be three-dimensional, porous, biocompatible, biodegradable, and susceptible to the growth of cells attached to it (Meng et al., 2014). Despite the many types of scaffolds available, both synthetic and natural (Meng et al., 2014), more and more information shows about using cadaver material to create scaffolds for implanted cells.

Gupta et al. presented a cell-free scaffold model made of postmortem collected goat lung tissue and used it for skin regeneration. In the first study, human skin-derived MSCs implanted on the scaffold showed adhesion, viability, proliferation, and expression of keratin 18 (specific for the skin), CD105, CD73, CD44 (specific for MSCs), which proved that they retained their original functions (Gupta et al., 2013a). In the second study, the scaffold from the goat’s lung tissue was further modified by placing an osteocyte on it and the subsequent modification with chitosan/nanohydroxyapatite. The cells showed higher proliferative capacity and expression of osteocalcin. Moreover, the MSCs present in the scaffold did not change the level of their differentiation, which accounted for their preserved viability (Gupta et al., 2013b). In the third study, also using goat lung tissue scaffold, Gupta et al. tested the scaffold modification with quercetin and nanohydroxyapatite (nHAp). The results showed that scaffolds with this modification are a better substrate for the growth of bone marrow MSCs and their ability to osteogenesis (Gupta et al., 2017). These three studies suggest that cadaver scaffolds may be a valuable tool in stem cell therapy in skin and bone regenerative medicine (Gupta et al., 2013a; Gupta et al., 2013b; Gupta et al., 2017).

Another approach is to take a scaffold from a deceased donor and implant it as a scaffold for autologous recipient cells. The scaffold in the form of a trachea fragment, which was a site for chondrocytes and epithelial cells grown from the recipient’s MSCs, successfully replaced the left bronchus of the recipient with end-stage bronchomalacia, ensuring proper airflow through the respiratory tract (Macchiarini et al., 2008). Importantly, the results of the 5-years follow up showed a significant improvement in the patient’s health, which allowed the authors o conclude that implanting a human trachea as a scaffold and a site for differentiating autologous stem cells is safe and might be more widely used in regenerative medicine (Gonfiotti et al., 2014). Scaffolds were also obtained from the canine larynx and used for the implantation of human epithelial cells collected postmortem (Moser et al., 2020). Another example of scaffold use is the extraction of decellularized scaffold from a human heart implanted with stem cells forming functional cardiocytes. Advances in this technology may eliminate the problem of the shortage of heart donors and the need for post-transplant immunosuppression, enabling the creation of personalized organs from postmortem material (Taylor, 2009). Ott et al. tried to obtain a functional heart scaffold from a rat cadaver. The experiment resulted in producing an organ that showed contractility and reactivity (Ott et al., 2008). A similar positive effect was obtained in rats for lungs scaffold isolated and subsequently transplanted (Ott et al., 2010). Maghsoudlou et al. presented an approach that combines postmortem stem cell harvesting and postmortem scaffold generation from tissues of the same species. Interestingly, these studies demonstrated the great potential of esophageal epithelial stem cells and the expression of their potential marker—CD34. They were isolated from the mice’s esophagus postmortem, and the best method of their isolation was the double incubation of the material with trypsin (Maghsoudlou et al., 2014).

## The Best Time and Place for Cadaveric Stem Cells (CaSCs) Harvesting

The changes that occur after death result from natural degradation processes and begin at the cellular level (Almulhim and Menezes, 2020). In addition to changes in gene expression in cells after death, the characteristics of the postmortem environment, creating a specific niche may also affect the properties of the stem cells harvested from cadavers. So far, it was possible to collect other than CaSCs types of cells from rats—for example, viable hepatocytes—even 8 h after death in the ischemic environment (Porretti et al., 2006), up to 24 h for chilled human cadavers, and even up to 27 h for mice chilled cadavers (Erker et al., 2010). In the most extreme case, it was possible to isolate progenitor cells for up to 7 days from the ischemic human liver (Stachelscheid et al., 2009). Moreover, HSCs showed high resistance to hypoxia. Since low oxygen levels are a characteristic feature of the postmortem environment and storage conditions (Michalova et al., 2011), the results suggested that maybe the postmortem environment does not affect the HSCs proliferation and viability. Studies on muscle stem cells showed they do not lose their properties d for up to 17 days postmortem (Latil et al., 2012), which contradicts the results of previous studies, where stem cells survived only a few days.

The time limit for cadaver stem cells harvesting depends on the type of cells and storage conditions. Moreover, since cadaver stem cells are a new research subject, many factors have yet to be discovered, as many cell types have not been studied under prolonged PMI.

Heparinization of the cadaver, when collecting other organs for transplantation, may also influence the viability of stem cells. Mesenchymal stem cells treated in this way retain viability and the ability to differentiate up to 7 days postmortem when stored at 4°C (Korchunjit et al., 2019). However, using liquid nitrogen to store the fragments of cadaveric arteries helps maintain the properties of MSCs for up to 5 years (Valente et al., 2014). On the other hand, a low temperature is a universal method of storage. Studies on mice showed that it is possible to generate neurospheres even 140 h postmortem when the material is stored at 4°C (Pruszak et al., 2009).

In the context of prolonged PMI, adipose stem cells present the unique ability to survive and differentiate even for 7 days postmortem. However, proper storage of the material is important to avoid the development of the rotting process (Saito et al., 2020). Studies on adipose stem cells’ cytokine profile indicate that blood cell growth factors or interleukin 7 (IL-7) play a significant role in hematopoiesis initiation. Moreover, keeping these cells in common cultures increases the number of myeloid and lymphoid progenitor cells (Kilroy et al., 2007). It is suggested that ADSCs are a promising source of multipotent stem cells compared to the usually used bone marrow (Zhu et al., 2008). A problem, which may also apply to cadaver stem cells, is their *in vitro* processing and decreasing ability to differentiate over time. Studies on live mice suggest that adding a low dose of antioxidants (melatonin and GSH) to an *in vitro* culture of ADSCs delays cell aging and helps maintain the ability to differentiate (Liao et al., 2019). High BMI value, hypertension, and acute carbon monoxide poisoning before donor’s death negatively affect the isolation and viability of ADSCs (Saito et al., 2020). Therefore it is necessary to compare the properties of stem cells originating from living and deceased donors to better understand their *in vitro* and *in vivo* capabilities when collected postmortem. Postmortem studies in rats showed no significant differences in the viability of NSCs up to 2 days after collecting them from living and cadaveric donors. After 2 days, the ability to create neurospheres significantly decreased in those collected from the cadavers (Xu et al., 2003). However, NSCs isolated from the human fetal spinal cord lose the proliferation capacity of primary neurospheres drastically around 12 h after death (Liu et al., 2006).

The research on the maximum PMI at which CaSCs can be harvested needs to continue. It is essential to know the limit for collecting viable and proliferating cells and the optimal conditions for storing them. Also, it would be helpful to know the gene expression in the postmortem environment and the survival limits in hypoxia and ischemia for specific types of stem cells.

## Limitations and Possibilities

Using cadaveric skin grafts (the most frequent procedure with dead donor material) is limited by the following instances: the unknown cause of donor’s death, diagnosed infectious diseases, cancer, collagen diseases, active dermatitis, current or finalized chemotherapy and radiotherapy, and immunosuppressant treatment during lifetime (Mosti et al., 2020). It seems necessary to thoroughly check how these factors affect stem cell transplants to create a specific protocol allowing for efficient qualification of dead donors based on autopsy results and disease history. Case report on scalp and dura mater fibroblasts transplants showed that the unattended death of the tissue donor, PMI length, and higher BMI levels were limiting factors for dermal cell cultures, opposed to dura mater fibroblasts case (Bliss et al., 2012). Studies on ADSCs in dolphins showed that those taken postmortem from adipose tissue had a significantly low level of viability, possibly related to tissue degradation and postmortem ischemia (Johnson et al., 2012). Importantly, in the case of some cells, such as retinal progenitor cells, the age and sex of the donor are not the limiting factors (Carter et al., 2007). The lack of such limitations increases the number of potential donors. The lack, or low amount, of relationship between postmortem lesions and gender, also increases the number of possible transplants (Prieto et al., 2004). However, stem cells obtained from adult donors, during life and postmortem, have a reduced potential for expansion and a limited differentiation potential compared to embryonic and fetal stem cells (Jarrige et al., 2021). In addition, therapy with somatic stem cells carries the risk of uncontrolled cell migration, tumorigenesis, or histocompatibility (Poulos, 2018). The problem could be solved by focusing on inducing pluripotency in somatic cells (such as fibroblasts). This approach can be widely used, among others, in modulating some neurodegenerative disorders (Hjelm et al., 2011; Bliss et al., 2012).

Moreover, it is necessary to test the effects of cadaver stem cell transplants to confirm their effectiveness *in vivo*. Over the years, many different methods have been developed for imaging and controlling transplanted stem cells. Labeling NSCs, and then monitoring their proliferation, migration, and integration into the tissue is possible thanks to real-time magnetic resonance imaging (MRI) (Obenaus et al., 2011). Many other methods of imaging are also applicable for transplanted cadaveric stem cells (Andrzejewska et al., 2015). All of them aim to assist researchers in assessing the effectiveness of the transplant.

In the face of numerous moral controversies caused by the isolation of stem cells from embryos, it is necessary to assess and examine the potential of stem cells obtained from adults, especially those isolated from postmortem material (Palmer et al., 2001). The amount of research on CaSCs compared to the widespread interest in stem cells is still too low to conclude on their therapeutic applications. However, the reports suggest that CaSCs retain viability and differentiation capacity even during PMI extended to several days. Tables 1 and 2 summarize the research on CaSCs harvesting from human cadavers (Table 1) and different animal species (Table 2) of different ages and PMI ranges.

**TABLE 1:** The summary of research on cadaveric stem cells (CaSCs) isolated from human cadavers.

Year	SCs type	PMI	Source of cells	Major findings	References
1999	NSCs	Not known	Fetal spinal cord	Differentiation into astrocytes and neurons; no oligodendrocytes; proliferation only to astrocytes in the presence of growth factors	Quinn et al. (1999)
2001	MSCs	<12 h	Knee synovium	Differentiation into an osteogenic, chondrogenic, and adipogenic line, and also occasionally into a myogenic line; cell potential not affected by donor age, passage, and cryopreservation	De Bari et al. (2001a)
2001	NSCs	2 h	Brain tissue of 11-weeks old male and temporal cortex of 27-years old male after resection	In the case of astrocyte and neurocyte cultures, progenitor cells were obtained in similar amounts from each sample; the highest concentration of NSCs in the hippocampus and the ventricular zone; a small number of oligodendrocyte cultures	Palmer et al. (2001)
2003	HSCs	<2 h	Pelvic bones, vertebral bodies of heparinized corpses	Bone marrow bags should be saturated with 20% oxygen; HSCs survive better when the bone marrow contains no red blood cells	Machaliński et al. (2003)
2004	RPCs (+BPCs)	>24 h	Retina and forebrain (premature newborns)	RPCs, unlike BPCs, express Dach1, Pax6, Six3, Six6, recoveri	Klassen et al. (2004)
2005	NPCs	<48 h	Retina and the flat part of the ciliary body (pars plana)	Generation of primary neurospheres, proliferation towards secondary neurospheres, neurons, photoreceptors and glial cells; RPCs positive for nestin, M neurofilament, rhodopsin, GFAB	Mayer et al. (2005)
2006	NSCs	2, 6, and 12 h at 4 °C	Fetal spinal cord	The most numerous cultures from the lumbosacral section a the spine; a large decrease in the ability to create neurospheres after 12 h	Liu et al. (2006)
2007	RPCs/NPCs	<44 h	Retina	Differentiation of neurospheres with different retinal cell lines: (glial, neuronal, and photoreceptors); PMI-dependent neurosphere formation rate	Carter et al. (2007)
2008	MSCs	<12 h	Periosteum of the proximal, medial part of the tibia and knee synovium	Periosteal MSCs had a higher osteogenic potential than those from the synovium; no differences between the sources in the MSCs phenotype; type I collagen and OPG as markers of osteogenic potential	De Bari et al. (2008)
2009	RPCs	12–18 weeks of gestation	Fetal retina	RPCs collected between 16 and 18 weeks of gestation have the best proliferative properties; differentiation towards photoreceptors; differentiation and expression of rhodopsin after transplant	Aftab et al. (2009)
2010	ADSCs	3 days before fixing + < 2 months before use	Adipose tissue from different parts of the body	Adipose tissue in the thoracic spine and lower abdomen are the richest source of ADSCs with the CD34 (+)/CD31 (-) phenotype	Kishi et al. (2010)
2011	RPCs	Not known	Iris pigment epithelium, ciliary body	Expression of RPCs markers: Pax6, Sox2, nestins; expression of markers of already differentiated cells: microphthalmia-associated transcription factor (MITF) and cytokeratin-19	Frøen et al. (2011)
2011	iPSCs	3–7 h	Skin fibroblasts	Presence of pluripotent ESCs markers; ability to proliferate towards neuronal and glial lines	Hjelm et al. (2011)
2011	HSCs	2, 6, and 12 h	Femur bone marrow	Progenitor cells (CFU-S/GM-CFC) present greater sensitivity to hypoxi than the stem cells; HSCs maintian viability up to 4 days at 4°C	Michalova et al. (2011)
2012	MSCs	Not known	Marginal zone of the cornea and sclera	Positive for CD29, CD34, CD39, CD73 and CD105; mediating immunosuppression together with the TCR receptor; constitutive secretion of TGFbeta1	Garfias et al. (2012)
2012	RPCs	24–48 h	Retinal pigment epithelium	Greater adhesion and faster proliferation of RPCs treated with amniotic fluid (increase in nuclear retinal progenitor markers: CHX10 and PAX6)	Sanie-Jahromi et al. (2012)
2012	Fibroblasts (iPSCs)	<46 h	Scalp skin, dura mater	Possibility to isolate fibroblasts suitable for generating iPSCs from both sources; faster proliferation for cultures of skin origin	Bliss et al. (2012)
2012	Muscle stem cells (MSCs)	6–17 days	Skeletal muscle	Stem cell survival up to 17 days for	Latil et al. (2012)
2014	MSCs	12 h + 5 years when strored in liquid nitrogen	Epiaortal area and the thoracic aorta	Strong proliferation in the first passages; expression of markers: CD44, CD73, CD90, CD105, HLA-G; expression of neuronal nestin; immunosuppressive properties against blood mononuclear cells	Valente et al. (2014)
2015	ADSCs	Not known	Fat tissue (abdominal wall)	ADSCs tend to cluster around vessels and in fibrous septa between adipose tissues; phenotype: CD34 (+), CD105 (+)	Di Taranto et al. (2015)
2015	MSCs	Not known	Hip bone (bone marrow)	Presence of CD105−, CD73^−^, CD44^−^ and CD90^−^, absence of CD45, CD34, CD14, CD11b, CD79, CD19, HLA-DR; transplanted cells regenere damaged skin of the patient	Mansilla et al. (2015)
2016	MSCs	Not known	Hip bone (bone marrow)	Expression of markers specific for MSCs; significant improvement after transplantation to a patient with necrotic changes in the leg (increase in beta-integrin and CRP levels)	Portas et al. (2016)
2017	MSCs	<24 h	Retina	Neuroprotective action (activation of AKT, ERK, and STAT3 pathways) of MSCs and the platelet-derived growth factor secreted by them on the ganglion cells of the retina	Shikh Alsook et al. (2015)
2020	ADSCs	<177 h (7 days)	Subcutaneous fat of the armpit	ADSCs can be successfully isolated up to 7 days postmortem; hypertension, carbon monoxide poisoning, and high BMI have a negative effect on the isolation and differentiation of ADSCs; ADSCs treated with collagen proliferate better	Saito et al. (2020)

Abbreviations: ADSCs, adipose delivered stem cells; BMI, body mass index; BPCs, brain progenitor cells; CRP, C-reactive protein; ESCs, embryonal stem cells; HSCs, hematopoietic stem cells; iPSCs, induced pluripotential stem cells; MSCs, mesenchymal stem cells; NPCs, neural progenitor cells; NSCs, neural stem cells; OPG, osteoprotegerin; PMI, postmortem interval; RPCs, retinal progenitor cells; SCs, stem cells

**TABLE 2:** The summary of research on cadaveric stem cells (CaSCs) isolated from dead animals.

Year	Species	SCs type	PMI	Source of cells	Major findings	References
1998	Mouse	NSCs	<140 h	Spinal cord, forebrain	The ability to form neurospheres declines with PMI; the generated neurospheres showed the ability to proliferate towards neurons and glial cells	Laywell et al. (1999)
2003	Rat	NSCs	<6 days	Forebrain, lateral ventricle and its striatum, subventricular zone	No differences between the neurospheres generated from living and deceased animals up to 2 days postmortem; greater number of neurospheres from cells of younger rats	Xu et al. (2003)
2007	Mouse (3-week old and newborn)	NSCs	<10 days	Vestibular and cochlear sensory epithelium (inner ear))	No differences in cells isolated at 5 and 10 days postmortem; possibility of isolating NSCs from both younger and older mice (3 weeks)	Senn et al. (2007)
2012	Horse (2–5 years old)	MSCs	Not known	Fat tissue, bone marrow, muscle tissue, periosteum	MSCs from bone marrow and adipose tissue have a lower osteogenic potential than those obtained from the periosteum and muscles	Radtke et al. (2013)
2012	Dolphin	ADSCs	6 h	Subcutaneous fat tissue	Cells collected from the cadaver are characterized by a much lower viability than those collected from a living donor	Johnson et al. (2012)
2012	Mouse	Muscle stem cells	<14 days	Skeletal muscle	Stem cell survival up to 14 days; Muscle Stem Cells transplanted into mice together with HSCs allows for strong regeneration; anoxia and hypoxia as factors of cell survival	Latil et al. (2012)
2015	Horse (18–20 years old)	MSCs	48–72 h	Suspensory ligament	Multipotency; the presence of markers CD73, CD90, CD105; dominance of cells with the appearance of fibroblasts; few cells with neuronal and glial features	Shikh Alsook et al. (2015)
2018	Goat, rat	NSCs	<56 h	Spinal cord	The maximum isolation time for human-size animal: 56 h, for a rat: 18 h; up to 1% of the cells were expressing the GD2 ganglyside and CD24 markers	Mikhailov and Sankai (2018)
2019	Horse (3–4 years old)	MSCs	4 h	Bone marrow	Positive for CD29 and CD90; expression of CD90, CD14, CD44 and POU5F1 genes; differentiation of MSCs + appropriate lines towards tenocytes; the transplant was successful	Korchunjit et al. (2019)

Abbreviations: ADSCs, adipose delivered stem cells; MSCs, mesenchymal stem cells; HSCs, hematopoietic stem cells; NSCs, neural stem cells; PMI, postmortem interval; SCs, stem cells

It is also necessary to test the ability of CaSCs to fulfill their role after transplantation into appropriate recipients. Further exploration and search for the PMI to which it is possible to collect viable and proliferating CaSCs is also needed. The same could be said about specific research on cancer stem cells and their properties and changes in the thanatotranscriptome of stem cells depending on PMI. Further research should also focus on modernizing the methods of cadaver storage to optimize them for CaSCs harvesting. Finally, developing a specific protocol for collecting, isolating, modifying *in vitro*, and using stem cells in specific clinical cases is of the utmost importance. However, any therapeutic options for CaSCs should be thoroughly investigated first regarding safety and confirmed scientific grounds to avoid risks of stem cell therapy (e.g., proliferative damage associated with transplanted cells) (Berkowitz et al., 2016).
